# Artificial Separations Versus Extraction of Six Year Molars

**Published:** 1876-08

**Authors:** G. R. Thomas

**Affiliations:** Detroit, Mich.


### ARTIFICIAL SEPARATIONS VERSUS EXTRACTION OF SIX YEAR MOLARS.

BY G. R. THOMAS, D. D. S., DETROIT, MICH.

I am very greatly obliged to Dr. L. C. Taylor for his criticism—that appears in the June number of the REGISTER—upon the article that appeared in the May nunber, entitled, "Artificial Separation of the Teeth as a Means of Prevention of Decay." He says, "while I endorse it heartily as far as it goes, I feel there is something lying far back practically of more value than the V shaped space for the preservation or the molars and bicuspids, viz., the timely and systematic extraction of the six year molars, which makes an artificial separation." I used to believe in the same thing, and still quite agree with the doctor this far, that it is much better to extract them than to do nothing at all, but in extracting does it not almost universally follow that nature's beautiful antagonizing model is completely broken up, so that the teeth never antagonize again as perfectly as before. Again, nature has provided a given number of teeth, not only for masticating purposes but to serve as feature builders, whereby the face receives its full measure of perfection.

I grant there are some cases that seem to be an abnormal production from the first, such as protruding lower jaw, or largely the reverse, where the timely extraction of a part of the six year molars would be a decided advantage in the way of expression to say nothing of the advantage gained in securing space. But I am decidedly of the opinion that the interests of the patient will be much better served, in the average mouth, by a thorough (and repeated if need be) perseverance of the V shaped openings than by the extraction of the six year molars.

It is true that in the case of the bicuspids and molars, their shape sometimes is such that they will come together again and require the second separation. But if the separations are given the proper angle, when they do come together, the points of contact will be near or at the necks, and the V will still remain.

By this mode of practice no teeth are sacrificed, the antagonizing model remains undisturbed and the features are allowed full development. But let it be understood that this mode of practice in order to be successful, *must* be commenced in season; and if I am asked what is meant by the "in season" my reply is, nothing can determine that except good judgment.

In the matter of separations also, it is not meant that rough ungainly, file cut separations are to be produced, but on the contrary, artistic, well shaped and highly polished surfaces and with the perfect appliances of the present day at our command, there is no trouble in producing such; thereby saving, as I believe, a far greater per centage of the teeth of the American poople, than by any other one mode of practice.

